# A Compact 2.3 μm DFB-Laser CO Sensor Using MPC-LITES for Real-Time Monitoring of Cigarette Smoke

**DOI:** 10.3390/s25226894

**Published:** 2025-11-12

**Authors:** Leqing Lin, Haoyang Lin, Guantian Hong, Jianfeng He, Lihao Wang, Ruobin Zhuang, Wenguo Zhu, Yongchun Zhong, Jianhui Yu, Huadan Zheng

**Affiliations:** College of Physics and Optoelectronic Engineering, Jinan University, Guangzhou 510632, China; llq119@stu2019.jnu.edu.cn (L.L.); linhaoyang12@gmail.com (H.L.); guantianhong@stu2024.jnu.edu.cn (G.H.); jnuhejian@stu.jnu.edu.cn (J.H.); eraserwang@163.com (L.W.); zrb618597@stu2020.jnu.edu.cn (R.Z.); zhuwg88@163.com (W.Z.); ychzhong@163.com (Y.Z.); kensomyu@gmail.com (J.Y.)

**Keywords:** optical gas sensing, light-induced thermoelastic spectroscopy, multi-pass cell, quartz tuning fork, carbon monoxide detection, indoor air quality monitoring

## Abstract

A compact and high-sensitivity carbon monoxide (CO) detection system based on multi-pass cell enhanced light-induced thermoelastic spectroscopy (MPC-LITES) was developed for real-time monitoring. A 2.3 μm distributed feedback (DFB) diode laser targeting the CO absorption line at 4300.699 cm^−1^ was employed, offering strong line intensity and minimal interference from H_2_O, CO_2_, NO_2_, and SO_2_. The optimal modulation depth of 0.76 cm^−1^ produced the maximum second harmonic (2*f*) signal. Experimental results demonstrated excellent linearity (R^2^ = 0.998) and a minimum detection limit of 230 ppb at 1 s, further reduced to 47 ppb at 367 s by Allan deviation analysis. Application tests were carried out for real-time monitoring of cigarette smoke in a 20 m^2^ indoor environment. Under closed conditions, the CO concentration rapidly increased to approximately 165 ppm, while in ventilated conditions, it peaked at 45 ppm and decayed quickly due to air exchange. The results confirm that the proposed MPC-LITES sensor enables accurate, real-time detection of transient CO variations, demonstrating strong potential for indoor air quality evaluation, environmental safety, and public health protection.

## 1. Introduction

Carbon monoxide (CO) is a colorless, odorless, and highly toxic gas that is widely generated from incomplete combustion processes, including industrial emissions, vehicle exhaust, and tobacco burning [1,2,3]. Long-term exposure to low CO concentrations can cause headaches, fatigue, and cognitive impairment, while acute exposure to high concentrations may lead to severe poisoning or even death [4,5,6]. According to the World Health Organization (WHO), more than 400,000 deaths worldwide each year are attributed to CO poisoning, with indoor air pollution identified as a major contributing factor [7,8,9,10,11]. In enclosed or poorly ventilated environments, the accumulation of CO from mainstream and secondhand smoke can rise to hazardous levels, posing serious health risks to non-smokers [12,13,14]. Therefore, the development of CO detection technologies that offer high sensitivity, fast response, real-time capability, and long-term stability is essential for environmental protection, public health, and industrial safety.

In recent years, numerous studies have emphasized the importance of indoor air pollution control and the advancement of optical gas sensing technologies for detecting hazardous gases [15,16,17,18]. These developments have further promoted the evolution of compact and high-sensitivity CO sensors designed for real-time applications. Conventional CO detection methods include electrochemical sensors, metal oxide semiconductor sensors, non-dispersive infrared (NDIR) spectroscopy, and gas chromatography–mass spectrometry (GC-MS) [19]. Electrochemical and MOS sensors feature low cost, compact size, and rapid response but suffer from limited lifetimes, strong cross-sensitivity to humidity and interfering gases, and poor stability [20,21]. Although recent progress has been made in improving MOS-based CO sensors, challenges such as humidity dependence and long-term drift still limit their suitability for continuous real-time monitoring [22,23]. NDIR sensors provide improved selectivity, yet their sensitivity is constrained by limited optical path lengths, generally achieving detection limits at the parts-per-million (ppm) level [24]. GC-MS enables highly sensitive and selective detection but requires bulky instrumentation, high cost, and offline analysis, making it unsuitable for real-time or portable applications [25,26]. Collectively, these limitations highlight the challenge of simultaneously achieving low detection limits, high selectivity, and robust stability in a single sensing platform.

Light-induced thermoelastic spectroscopy (LITES), an emerging optical gas sensing technique, has attracted increasing attention in recent years due to its high sensitivity, compact configuration, and immunity to environmental noise [27,28,29,30,31]. In LITES, the thermoelastic response generated by molecular absorption is transduced by a quartz tuning fork (QTF) into an electrical signal, enabling sensitive and stable detection [32,33,34,35,36,37,38,39,40]. However, the relatively short optical path in conventional LITES setups limits the absorption strength and thus restricts detection sensitivity for trace gases [41,42,43,44,45]. Incorporating a multi-pass cell (MPC) can effectively address this issue by extending the optical path length to the meter scale, significantly amplifying the thermoelastic signal [46,47,48,49,50].

In this work, a multi-pass cell enhanced light-induced thermoelastic spectroscopy (MPC–LITES) system was proposed and experimentally demonstrated for real-time and highly sensitive detection of CO. A 2.3 μm distributed feedback (DFB) diode laser was employed as the excitation source, targeting the CO absorption line at 4300.699 cm^−1^, which exhibits strong line intensity and negligible interference from H_2_O, CO_2_, NO_2_, and SO_2_. The optical path is extended to ~8 m by an MPC. The detailed spectral line selection, laser characterization, and system configuration are presented, in which the system was optimized to achieve maximum signal stability and detection sensitivity. The optimized MPC-LITES system achieved a minimum detection limit of 230 ppb at 1 s and 47 ppb at 367 s. To verify its practical applicability, the system was applied to indoor CO monitoring during cigarette combustion under both closed and ventilated conditions. Significant differences in CO accumulation and decay behavior were clearly observed, with concentrations rising to 165 ppm in a closed room and only 45 ppm under ventilation. These results demonstrate that the developed MPC-LITES sensor enables accurate and real-time quantification of CO in dynamic indoor environments, providing a compact, stable, and field-deployable solution for air-quality monitoring, environmental assessment, and health-risk prevention.

## 2. Experimental Setup

### 2.1. Spectral Line Selection

**Figure 1:**
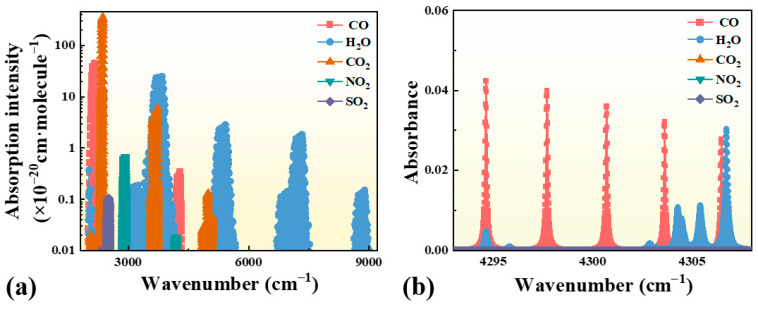
(**a**) Simulated absorption line strengths of CO, H_2_O, CO_2_, NO_2_, and SO_2_ in the 2000–9000 cm^−1^ range at 300 K, based on the HITRAN 2020 database. (**b**) Simulated molecular absorption cross-sections of CO, H_2_O, CO_2_, NO_2_, and SO_2_ in the 4293–4308 cm^−1^ range at 300 K, 1 atm, and a 100 cm optical path, with mixing ratios of 0.001 for CO and 0.2 for interfering gases.

The selection of an appropriate spectral line is a critical prerequisite for accurate in situ CO detection using LITES, as it directly determines the system’s sensitivity, interference suppression capability, and practical applicability. Figure 1a,b illustrate the complete line-selection process, from identifying the spectral region to determining the specific absorption transition.

As shown in Figure 1a, the absorption line strengths of CO, H_2_O, CO_2_, NO_2_, and SO_2_ were simulated using the HITRAN 2020 database at 300 K [51]. The results indicate that CO exhibits two prominent absorption bands, located near 2.3 μm and 4–5 μm. The 2.3 μm band was selected for practical implementation because distributed feedback (DFB, Healthy Photon, Ningbo, China) diode lasers operating in this region are compact, cost-effective, and easily integrated with multi-pass optical configurations, in contrast to those in the mid-infrared (4–5 μm) range. In addition, the 2.3 μm region provides practical advantages such as room-temperature operation, mature and low-cost optical components, and the availability of fiber-coupled DFB lasers, which together enable compact and robust system integration. To further identify the optimal CO transition line, Figure 1b shows the simulated molecular absorption cross-sections of CO, H_2_O, CO_2_, NO_2_, and SO_2_ within the range of 4293–4308 cm^−1^ under standard conditions (300 K, 1 atm) and a 100 cm optical path. The gas mixing ratios were set to 0.001 for CO and 0.2 for the interfering species, ensuring comparable spectral visibility. The results reveal that the CO transitions at 4297.705 cm^−1^ and 4300.699 cm^−1^ are relatively isolated and show negligible spectral overlap with absorptions from the listed interfering gases.

Based on broadband temperature-dependent analyses of the CO lines in the 2.3 μm region reported by Ruan et al. [52], the 4300.699 cm^−1^ transition was finally selected. This line has an absorption strength on the order of 10^−21^ cm/molecule and exhibits excellent stability of broadening parameters from 300 to 1100 K, making it a robust and temperature-insensitive choice for practical CO monitoring.

### 2.2. Characteristics of the 2.3 μm DFB Diode Laser

**Figure 2:**
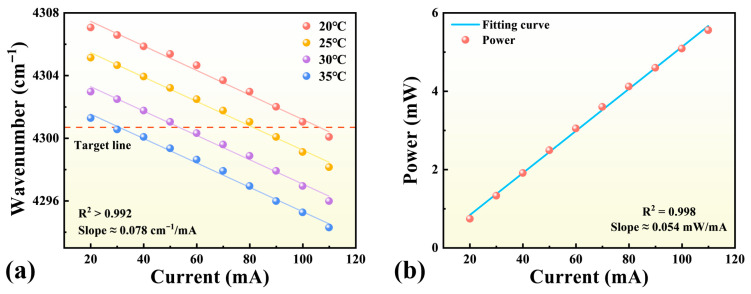
Characteristics of the 2.3 μm DFB diode laser. (**a**) Wavelength versus injection current at TEC temperatures of 20 °C, 25 °C, 30 °C, and 35 °C. (**b**) Output power as a function of injection current, showing an approximately linear growth.

The characteristics of the 2.3 μm DFB diode laser used in the LITES system are shown in Figure 2. Prior to the experiments, wavelength calibration was performed using a Bruker Fourier transform infrared spectrometer (VERTEX 70, Bruker Optik GmbH, Ettlingen, Germany) to ensure precise wavelength tuning and alignment with the target CO transition. The laser emission wavelength was tuned by adjusting the thermoelectric cooler (TEC) temperature and injection current through a commercial controller (CLD1015, Thorlabs, Inc., Newton, NJ, USA).

As presented in Figure 2a, the output wavelength exhibits a clear linear dependence on injection current at TEC temperatures of 20, 25, 30, and 35 °C. The fitted results show R^2^ > 0.992 with an average slope of 0.078 cm^−1^/mA, confirming excellent wavelength tuning linearity. After optimization, when the TEC temperature was set to 20.5 °C and the injection current to 97 mA, the emission wavelength stabilized at 2325.2 nm, corresponding to the selected 4300.699 cm^−1^ CO absorption line. At this transition, the absorption line strength is approximately 2.63 × 10^−21^ cm/molecule. Figure 2b shows the output power characteristics, which increase nearly linearly with injection current, exhibiting R^2^ = 0.998 and a slope of 0.054 mW/mA, reaching approximately 5 mW at 97 mA. These results satisfy the requirements for high-sensitivity gas detection.

### 2.3. Experimental Setup of the MPC–LITES System

**Figure 3:**
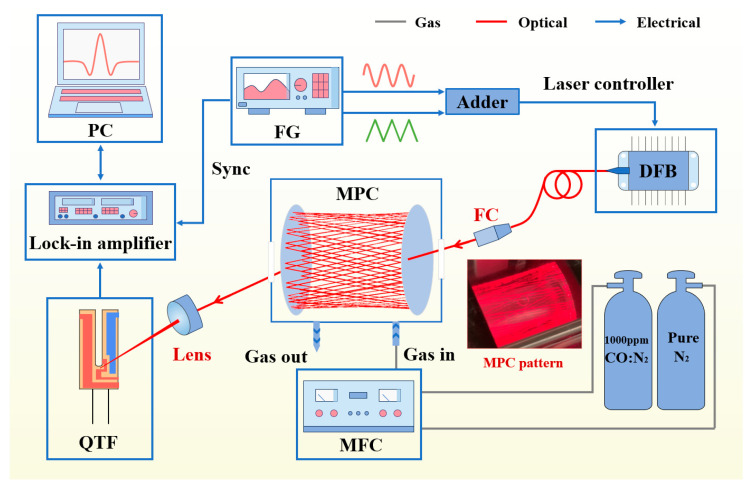
Schematic of the experimental setup for the MPC–LITES CO detection system. PC: personal computer, QTF: quartz tuning fork, FG: functional generator, MPC: multi-pass cell, MFC: mass flow controllers, FC: fiber collimator, DFB: distributed feedback laser. Red: optical path, gray: gas path, blue: electronic circuit, pink curve: sine signal, green curve: ramp signal.

The experimental configuration of the LITES-based CO detection system is illustrated in Figure 3. A dual-channel arbitrary function generator (Tektronix AFG3102C, Beverton, OR, USA) generated sinusoidal and ramp signals, which were superimposed by an adder and applied to the laser controller (CLD1015, Thorlabs, Inc., Newton, NJ, USA). The controller simultaneously adjusted the injection current and TEC temperature of the DFB laser to achieve wavelength modulation around 2325.2 nm (≈4300.699 cm^−1^).

The modulated laser beam was delivered through an optical fiber and collimated into an MPC comprising two silver-coated spherical mirrors (diameter: 50 mm, radius of curvature: 300 mm) separated by 5 cm. With a mirror reflectivity of 98% in the 2–20 μm range, the effective optical path length was extended to approximately 8 m through 160 reflections, thereby enhancing the CO absorption signal. Gas mixtures of CO and N_2_ were regulated by mass flow controllers (MFCs, Alicat, Tucson, AZ, USA) to achieve desired concentrations before entering the MPC.

The transmitted laser was detected by QTF. The resulting LITES signal was processed by a lock-in amplifier (SR830, Sunnyvale, CA, USA) performing 2*f* demodulation, and the demodulated signal was acquired by a data acquisition (DAQ) system via LabVIEW (National Instrument, Austin, TX, USA). The function generator also provided synchronization signals to ensure phase stability. To minimize external disturbances, the MPC module was sealed and maintained at 32 °C, with all measurements conducted under standard atmospheric pressure.

### 2.4. Frequency Response of the Quartz Tuning Fork

**Figure 4:**
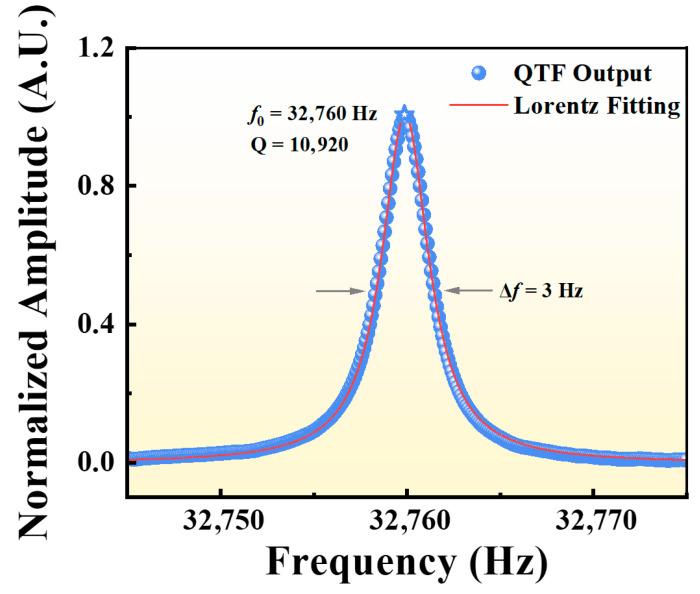
Frequency response curve of the QTF under atmospheric pressure, obtained by electrical excitation. The experimental data (blue dots) are fitted with a Lorentzian function (red line).

Figure 4 shows the frequency response of the commercial QTF, along with a Lorentzian fit to the measured data. The measurement was performed under atmospheric pressure using electrical excitation. The QTF exhibits a resonance frequency (*f*_0_) of 32,760 Hz and a full width at half maximum (FWHM) bandwidth of Δ*f* = 3 Hz, corresponding to a quality factor Q = *f*_0_/Δ*f* = 10,920. This high Q-factor reflects excellent mechanical resonance and energy confinement, ensuring efficient signal amplification and noise suppression in the LITES-based CO detection system.

## 3. Results and Discussion

### 3.1. Optimization of the Modulation Depth

**Figure 5:**
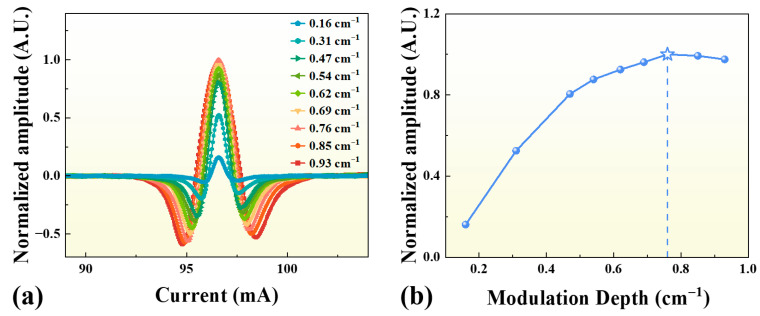
Optimization of modulation depth for the LITES-based CO detection system. (**a**) Normalized 2*f* signal amplitude versus injection current at different modulation depths. (**b**) Dependence of normalized signal amplitude on modulation depth, showing a maximum (star sign) at 0.76 cm^−1^, which was chosen as the optimal modulation depth for subsequent measurements.

In wavelength modulation spectroscopy (WMS) combined with 2*f* demodulation, the modulation depth is a critical parameter that directly determines the signal quality and sensitivity of the LITES-based sensing system. Here, the modulation depth (in cm^−1^) is defined as the amplitude of the laser wavenumber modulation caused by the sinusoidal modulation of the injection current. Figure 5 presents the optimization process and results for the laser wavelength modulation depth.

As illustrated in Figure 5a, both the shape and amplitude of the normalized 2*f* signal vary significantly with modulation depth, exhibiting distinct differences in peak-to-peak response as the injection current is scanned. The quantitative dependence shown in Figure 5b reveals that the normalized signal amplitude initially increases with increasing modulation depth, reaches a maximum at 0.76 cm^−1^, and then gradually declines as the modulation depth continues to increase.

This trend indicates that the thermoelastic signal strength of the LITES system is highly sensitive to the modulation depth. A modulation depth of 0.76 cm^−1^ was therefore selected as the optimal operating parameter to ensure strong and stable sensor performance in subsequent measurements.

### 3.2. Sensor Response and Calibration Linearity

**Figure 6:**
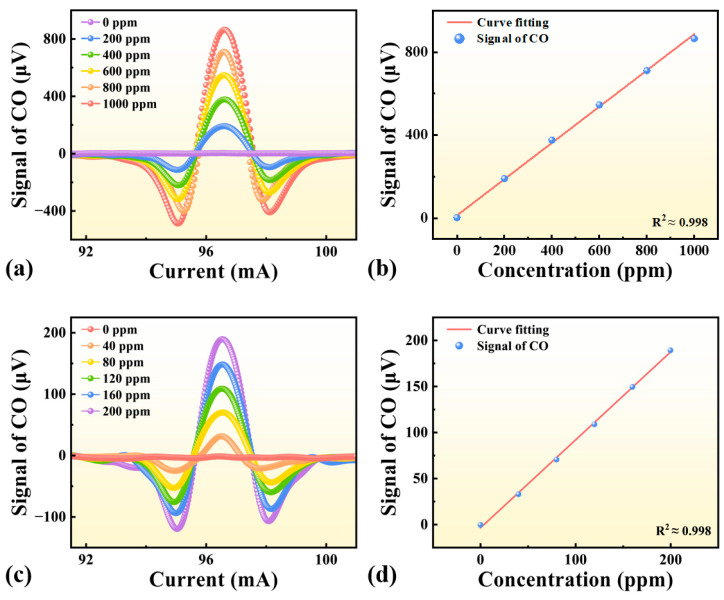
Response characteristics of the LITES-based CO sensor at different concentrations. (**a**) 2*f* signal profiles as a function of injection current for CO concentrations of 0–1000 ppm. (**b**) Linear relationship between 2*f* peak amplitude and CO concentration (R^2^ = 0.998). (**c**) 2*f* signal profiles at lower CO concentrations (0–200 ppm). (**d**) Linear fitting of the low-concentration calibration data (R^2^ = 0.998), demonstrating consistent linearity and sensitivity over the entire measurement range.

The response characteristics of the developed sensor to various CO concentrations are shown in Figure 6. During the experiments, the total gas flow was maintained at 200 sccm, and CO concentrations were precisely generated using calibrated mass flow controllers. As presented in Figure 6a, the amplitude of the 2*f* signal increases monotonically with CO concentration from 0 to 1000 ppm, demonstrating a strong correlation between signal strength and concentration. The 2*f* peak value reached 864.5 μV at 1000 ppm CO. The corresponding calibration curve in Figure 6b exhibits excellent linearity with a determination coefficient of R^2^ = 0.998.

To further evaluate the performance at lower concentrations, additional calibration experiments were performed in the range of 0–200 ppm, as shown in Figure 6c,d. The 2*f* signal maintains clear proportionality to the CO concentration, and the fitted curve again yields R^2^ = 0.998, confirming reliable sensor response and good linearity across both high and low concentration ranges.

This high linearity confirms the reliable quantitative detection capability of the proposed sensor and underscores its potential for accurate CO monitoring across a wide concentration range.

### 3.3. Detection Limit and Allan Deviation Analysis

**Figure 7:**
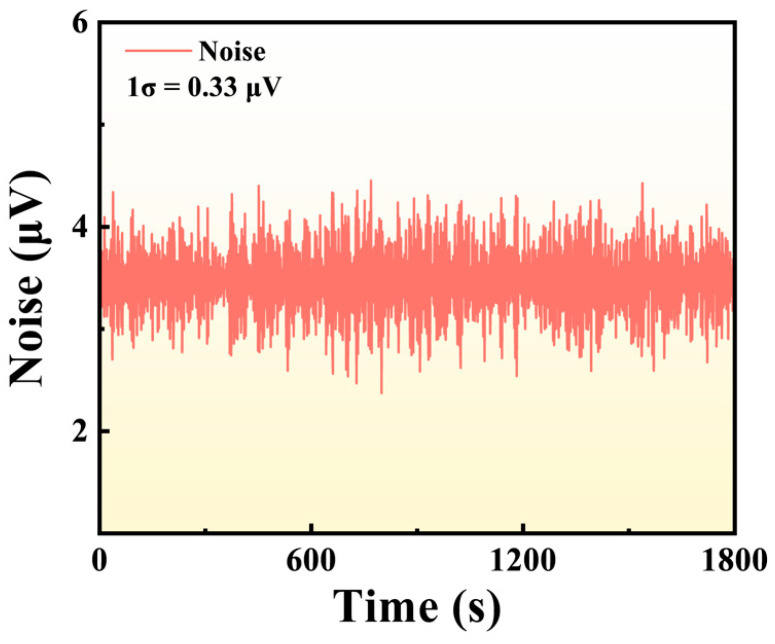
Noise characteristics of the LITES-based CO sensor under pure N_2_ flow at 200 sccm over an 1800 s period.

To assess the intrinsic noise characteristics of the optimized sensor, pure N_2_ was continuously introduced into the gas cell at a flow rate of 200 sccm. As shown in Figure 7, the sensor output exhibits random fluctuations during a continuous 1800 s acquisition period, indicating stable baseline performance over an extended duration. Statistical analysis of the baseline signal yields a standard deviation of 0.33 μV. Based on this noise level, the signal-to-noise ratio (SNR) was calculated as 590 corresponding to a minimum detection limit (MDL) of 0.33 ppm. These results demonstrate that the LITES-based CO sensor achieves both low background noise and high sensitivity, enabling the reliable detection of trace CO levels.

**Figure 8:**
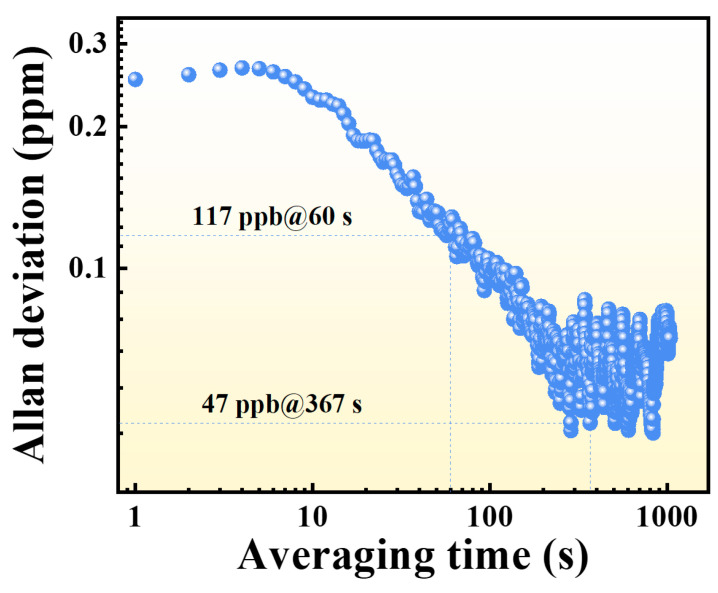
Allan deviation analysis of the LITES-based CO detection system.

To further evaluate long-term stability and ultimate detection performance, Allan deviation analysis was conducted [45,46,53]. During the test, the laser wavelength was fixed at the CO absorption peak, while pure N_2_ was continuously supplied for over two hours. As shown in Figure 8, the Allan deviation decreases by increasing averaging time up to a certain point before rising again. At an averaging time of 1 s, the Allan deviation was 230 ppb, which decreased significantly to 117 ppb at 60 s. The minimum Allan deviation of 47 ppb was obtained at an averaging time of 367 s, representing the optimal integration period for the system.

These results confirm that the developed sensor exhibits excellent long-term stability and ultra-low noise, achieving CO detection with sub-ppm precision and even sub-100 ppb resolution.

### 3.4. Real-Time Application in Indoor CO Monitoring

**Figure 9:**
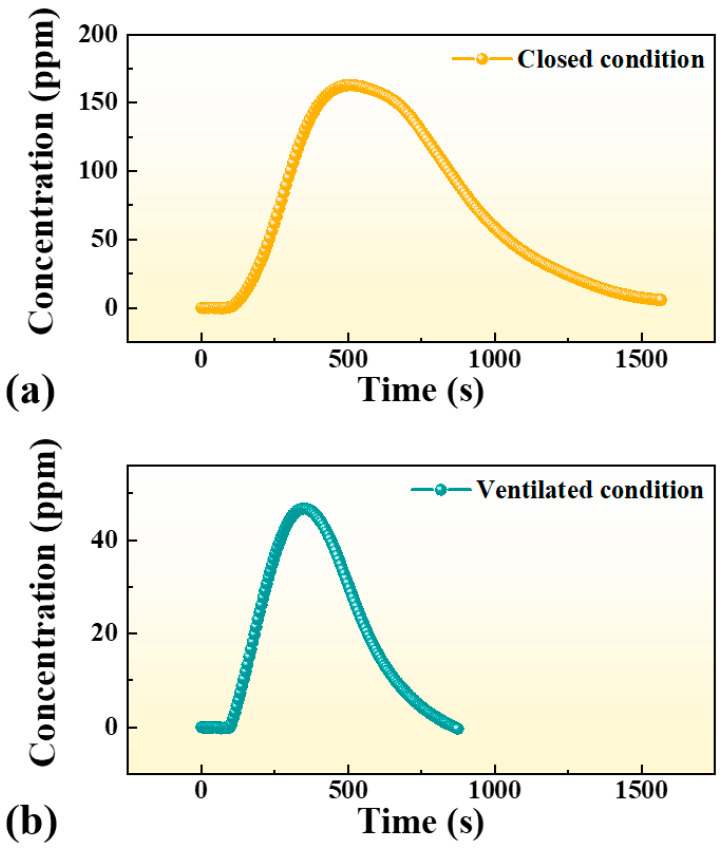
Real-time CO concentration monitoring of cigarette smoke using the MPC–LITES sensor under (**a**) closed and (**b**) ventilated indoor conditions.

To assess the sensor’s practical applicability, real-time monitoring experiments were performed during cigarette combustion within an indoor space of approximately 20 m^2^-representative of a typical small room or office environment. As shown in Figure 9, two environmental scenarios were evaluated: (i) a closed condition without ventilation, and (ii) a ventilated condition with natural airflow through an open window.

Each experiment began with a 100 s background measurement to establish the baseline CO concentration. At t = 100 s, a cigarette was ignited, and CO concentration was continuously recorded during combustion and subsequent dispersion. Under the closed condition, the CO concentration initially remained near zero, then rose sharply after ignition, reaching a peak of approximately 165 ppm within 400 s. The concentration subsequently decreased slowly due to limited diffusion and minor air leakage. Although the overall exposure duration did not reach one hour, the CO level exceeded the WHO one-hour guideline value of 26 ppm for about 900 s [54], and the instantaneous peak was much higher. Such transient but intense accumulation underscores the potential health risks posed by CO buildup in unventilated spaces. In contrast, under the ventilated condition, the peak CO concentration was significantly lower—approximately 45 ppm—and decayed rapidly as air exchange facilitated smoke dispersion. The faster decline and reduced peak clearly demonstrate the critical role of ventilation in mitigating indoor CO accumulation.

Overall, these application tests confirm that the MPC-LITES sensor enables high-sensitivity, real-time tracking of transient CO variations in realistic environments. The system effectively captured both the accumulation and dissipation dynamics of smoke-derived CO, demonstrating its strong potential for applications in indoor air quality assessment, health risk evaluation, and environmental safety monitoring.

## 4. Conclusions

A compact and highly sensitive CO sensor based on multi-pass cell-enhanced light-induced thermoelastic spectroscopy (MPC-LITES) was developed and systematically evaluated. Using a 2.3 μm DFB diode laser targeting the 4300.699 cm^−1^ CO line, the system achieved precise wavelength modulation and strong absorption response. The optimized modulation depth of 0.76 cm^−1^ produced the highest 2*f* signal amplitude. The sensor exhibited an excellent linear response (R^2^ = 0.998) across 0–1000 ppm and achieved a minimum detection limit of 230 ppb at 1 s and 47 ppb at 367 s, with outstanding noise suppression and long-term stability verified by Allan variance analysis.

In practical indoor experiments, the developed system successfully captured the dynamic variation in CO concentration generated by cigarette combustion. Under unventilated conditions, the CO level rapidly rose to approximately 165 ppm, remaining above the WHO one-hour guideline level of 26 ppm for roughly 900 s before gradually declining. With adequate ventilation, the peak concentration dropped to about 45 ppm, and dissipation was significantly faster. These results confirm that the MPC-LITES sensor provides real-time, low-noise, and high-resolution CO monitoring, demonstrating strong potential for indoor air-quality assessment, health-risk evaluation, and environmental-safety applications.

## Data Availability

The data presented in this study are available on request from the corresponding authors.
